# Host Acceptance and Plant Resistance: A Comparative Behavioral Study of *Myzus persicae* and *Acyrthosiphon pisum*

**DOI:** 10.3390/insects12110975

**Published:** 2021-10-28

**Authors:** Yi-Syuan Jhou, Sushanthi Poovendhan, Li-Hsin Huang, Chi-Wei Tsai

**Affiliations:** 1 Department of Entomology, National Taiwan University, Taipei 106, Taiwan; r07632002@ntu.edu.tw (Y.-S.J.); sushanthip96@gmail.com (S.P.); 2 Pesticide Application Division, Taiwan Agricultural Chemicals and Toxic Substances Research Institute, Taichung 413, Taiwan; lhhuang@tactri.gov.tw

**Keywords:** aphid, electrical penetration graph, behavior, host plant

## Abstract

**Simple Summary:**

Aphids are one of the most destructive insect pests worldwide. The green peach aphid (*Myzus persicae*) feeds on a broad range of plants, whereas the pea aphid (*Acyrthosiphon pisum*) only feeds on legumes. In this study, these two aphid species were used to investigate host acceptance and plant resistance to aphid feeding. Experiments on host plant preference and aphid performance (with regard to survival, development, and fecundity) confirmed that rape (*Brassica rapa*) is a suitable host and that faba bean (*Vicia faba*) is a poor host for the green peach aphid; for the pea aphid, faba bean is a suitable host, whereas rape is a nonhost. The probing and feeding behavior of these two aphid species on rape and faba bean was examined, and the results demonstrated the feeding preferences of these two aphid species. The green peach aphid had difficulty ingesting the phloem sap of faba bean. For the nonhost, the pea aphid spent relatively little time on mesophyll probing and did not achieve phloem sap ingestion. Furthermore, the effects of the probing and feeding behavior of specialist and generalist aphids on the spread of plant diseases caused by viruses were discussed.

**Abstract:**

Aphids are prominent phloem-feeding insect pests. *Myzus persicae* and *Acyrthosiphon pisum* are generalist and specialist species, respectively. In this study, these two aphid species were used to investigate host acceptance and plant resistance to aphid feeding. *M.* *persicae* survived and reproduced on rape (*Brassica rapa*), but few individuals (9%) survived on faba bean (*Vicia faba*). *A.* *pisum* survived and reproduced on faba bean, but no *A.* *pisum* survived on rape. The probing and feeding behavior of *M. persicae* and *A. pisum* on rape and faba bean was examined using an electrical penetration graph (EPG) technique. The results demonstrated the feeding preferences of these two aphid species. The EPG results suggest that the resistance of faba bean to *M. persicae* and that of rape to *A. pisum* are likely residing in the phloem and mesophyll tissues, respectively. Due to the distinct probing and feeding behaviors, specialist and generalist aphids would have different impacts on the epidemiology of plant viral diseases. The findings can be applied to the management of viral diseases transmitted by specialist or generalist aphids in crop production.

## 1. Introduction

Aphids, prominent phloem-feeding pests, are distributed worldwide. The green peach aphid, *Myzus persicae* (Sulzer), is a generalist species that mainly feeds on plants in the Brassicaceae, Solanaceae, and Fabaceae families [1,2]. The pea aphid, *Acyrthosiphon pisum* (Harris), is a specialist species, and its host range is limited to the Fabaceae family [3]. Aphid probing and feeding can cause direct injury to plant tissues, inducing serious tissue distortion, and indirectly transmit viruses to host plants [4,5].

The interaction between aphids and plants can be evaluated by examining the host preference, performance, and feeding behavior of aphids. Despite being a generalist species, *M. persicae* has a preference for various host plants. Gynoparous, oviparous, and apterous *M. persicae* prefers radish (*Raphanus sativus*) to peach (*Prunus persica*) [6]. Regarding legume crops, *M. persicae* prefers dwarf bean (*Phaseolus vulgaris*) and pea (*Pisum sativum*); whereas *A. pisum* prefers faba bean (*Vicia faba*) and clover (*Trifolium subterraneum*) [7]. Studies have reported that *A. pisum* exhibited the highest survival rate, highest fecundity, and heaviest body weight on faba bean [8,9]. *M. persicae* reared on faba bean exhibited a lower population growth rate than when reared on oilseed rape (*Brassica napus*) [10]. Even though the generalist *M. persicae* can feed on faba bean, it has difficulty ingesting the phloem sap [11]. Similar to *A. pisum* ssp. *destructor* on *Ononis repens, Sarothamnus scoparius*, and *Vicia cracca* [12], aphids cannot survive and reproduce on their nonhost plants [13,14,15].

After landing on a plant, an aphid assesses the plant’s surface characteristics and commences test probing to examine its inner composition [16]. If the aphid accepts this host candidate, it initiates prolonged feeding through the ingestion of phloem sap to obtain nutrients. Otherwise, the aphid abandons the plant to search for another. The process of test probing and prolonged feeding is closely related to plant damage and plant virus transmission [16,17]. To analyze the probing and feeding behavior of sap-feeding insects in depth, the electrical penetration graph (EPG) technique can be employed [18,19]. This approach is mainly applied in studying the probing and feeding activities of hemipterans (e.g., aphids, whiteflies, planthoppers, leafhoppers, and psyllids) [20,21,22,23].

Aphid test probing can occur in both host and nonhost interactions [16,17,22,24]. Test probing is responsible for the high transmission rate of nonpersistent viruses (e.g., cucumoviruses and potyviruses) by aphids [4,17,25,26]. Following acceptance of a host plant after test probing, aphids proceed to prolonged feeding to ingest phloem sap. When aphids feeding on host plants, phloem salivation is followed by phloem sap ingestion. This process is related to the inoculation and acquisition of semipersistent viruses (e.g., closteroviruses), circulative-nonpropagative viruses (e.g., luteoviruses), and circulative-propagative viruses (e.g., rhabdoviruses) transmitted by aphids [4,17,27,28]. Specialist and generalist aphids would have different impacts on the epidemiology of plant viral diseases.

The objective of this study was to investigate the host acceptance of aphids and plant resistance to aphid feeding. The aphid species of interest were *M. persicae* and *A. pisum*. Host plant preference and aphid performance experiments confirmed that rape (*B. rapa*) and faba bean (*V. faba*) are suitable and poor hosts, respectively, for *M. persicae.* As for *A. pisum*, faba bean is a suitable host, whereas rape is a nonhost. The aphid’s probing and feeding behavior examined using the EPG were compared to determine the mechanisms of host acceptance and plant resistance. The findings can be applied in managing the viral diseases transmitted by specialist or generalist aphids in crop production.

## 2. Materials and Methods

### 2.1. Aphids and Plants

*M. persicae* and *A. pisum* were reared on rape (*B. rapa* cv. KY Early) and faba bean (*V. faba* cv. KY Broad Bean) seedlings, respectively, and enclosed in 2-L beakers covered with insect-proof nets (109 mesh/in) in a climate chamber at 25 °C under a photoperiod of L:D 16:8 h. Rape and faba bean seedlings were grown from seeds (Known-You Seed, Kaohsiung, Taiwan) in a mixture of peat moss and vermiculite (1:1) and fertilized weekly with HYPONeX No. 2 Fertilizer (Hyponex, Marysville, OH, USA). All the plants were cultivated in a climate chamber at 28 °C under a photoperiod of L:D 16:8 h. Both rape and faba bean seedlings with 4–5 true leaves were used.

### 2.2. Host Plant Preference

The preference of aphids to candidate plants was examined through choice experiments. In this case, 10 apterous adults of *M. persicae* or *A. pisum* were confined in a petri dish (dia. 35 mm) and positioned in the center of a transparent acrylic cage (21 × 14 × 5 cm height). A detached young and fully expanded leaf of the rape and faba bean seedlings was attached to the edge of the petri dish. The experiment began with the opening of the petri dish to release the caged aphids. The numbers of aphids that settled on the rape and faba bean leaves after 1 and 3 h were recorded. Both aphids and plant leaves were used only once. Each experiment was conducted 10 times.

### 2.3. Aphid Performance

The survival, developmental time, adult body weight, and fecundity of *M. persicae* and *A. pisum* were determined to evaluate their performance on the plants. First-instar nymphs were produced by five apterous adults that had been reared for 2 days on their original host plant. The newborn nymphs were then transferred to test plants using a fine brush. The experiments were conducted with the following four treatment groups: *M. persicae* reared on rape, *M. persicae* reared on faba bean, *A. pisum* reared on rape, and *A. pisum* reared on faba bean. In this case, 10 first-instar nymphs were enclosed with one leaf of each test plant in a gauze bag (8 × 15 cm, 109 mesh/in). The survival rate was calculated daily, and the time of development from first instar to adult was recorded. The body weight of the newly developed adults was measured using a precision balance, and then each aphid was placed in a petri dish (dia. 90 mm) with a detached rape or faba bean leaf and a wet cotton pledget to maintain moisture for progeny production. Fecundity was evaluated as the total number of progenies produced within 5 days. Each experiment was repeated 10 times.

### 2.4. Probing and Feeding Behavior

The EPG technique was employed to monitor the probing and feeding behavior of *M. persicae* and *A. pisum* on the rape and faba bean seedlings. The experiments were conducted with the following four treatment groups: *M. persicae* on rape, *M. persicae* on faba bean, *A. pisum* on rape, and *A. pisum* on faba bean. Every examined insect and plant was used only once. Each experiment was replicated 20 times.

### 2.5. Electrical Penetration Graph (EPG)

A Giga-8 DC EPG system (EPG Systems, Wageningen, The Netherlands) was used to monitor the probing and feeding behavior of the aphids. Each aphid was attached to a thin gold wire (dia. 12.5 μm) by using a conductive silver glue, LOCTITE EDAG 503 62% E&C (Ladd Research Industries, Williston, VT, USA), that was smeared on aphid’s dorsal thorax. The other end of the gold wire was adhered to a copper nail using the same glue. Wired aphids had 15 min to adapt to the wiring before being placed on the examined plants. Subsequently, the copper nail connected to the wired aphid was fitted into the input of the EPG probe, and the substrate electrode was inserted into the moist soil of the potted plant. EPG monitoring was conducted at 25 °C inside a Faraday cage to shield from external electrical noise. Waveform events were recorded using Stylet+ software (EPG Systems) for 8 h. Aphid probing and feeding waveforms were identified manually with references to Tjallingii [19,29] and were labeled using Stylet+. The EPG parameters were then calculated using Excel Workbook (Microsoft, Redmond, WA, USA) for automatic parameter calculation [30]. Four non-phloem parameters (total duration of not probing, number of probes, total duration of the pathway phase, and number of potential drops) were calculated and analyzed. Six phloem-related parameters (proportion of reaching phloem sap ingestion, time from first probe to first phloem salivation, time from that probe to first phloem salivation, total duration of phloem salivation, number of phloem salivations followed by sap ingestions, and total duration of phloem sap ingestion) and two xylem-related parameters (number and total duration of xylem ingestions) were also calculated and analyzed. Regarding continuous sap ingestion, the sap ingestion period was calculated until the end of 8-h EPG monitoring and may have been underestimated.

### 2.6. Statistical Analyses

The percentage of aphids that settled on the test plants was analyzed using the Mann-Whitney *U* test, because the data were not normally distributed (Supplementary Materials). This test was also applied to assess the aphid’s survival rate, developmental time, and body weight. The EPG parameters were analyzed using the χ^2^ test and *t* test for normally distributed data and the Mann-Whitney *U* test for non-normally distributed data. Analyses were performed using IBM SPSS Statistics for Windows, version 19 (IBM Corp., Armonk, NY, USA).

## 3. Results

### 3.1. Host Plant Preference

Choice experiments were conducted to examine the host plant preference of *M. persicae* and *A. pisum*. Approximately 90% of *M. persicae* preferred rape over faba bean as measured 1 h and 3 h after aphid release (Mann-Whitney *U* test, *p* < 0.05; Table 1). Not all *A. pisum* chose either plant; about 50–60% of *A. pisum* remained in place and did not investigate the plant leaves. However, *A. pisum* still preferred faba bean over rape (Mann-Whitney *U* test, *p* < 0.05; Table 1). The results suggest that rape and faba bean are preferred and non-preferred hosts for *M. persicae*, respectively, while faba bean and rape are preferred and non-preferred hosts for *A. pisum*, respectively.

### 3.2. Performance of M. persicae

**Figure 1:**
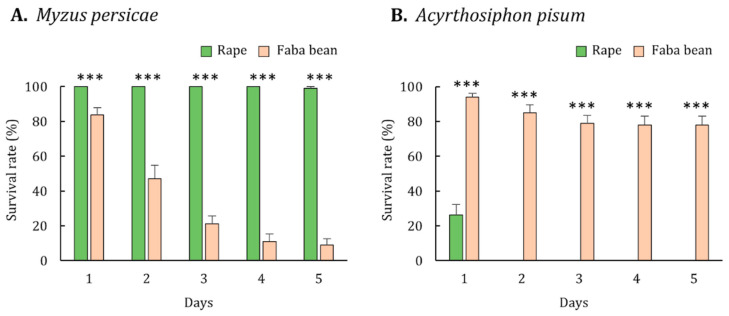
Survival rate of (**A**) *Myzus persicae* and (**B**) *Acyrthosiphon pisum* reared on rape and faba bean. Error bars represent the standard errors of the means. Triple asterisks indicate significant differences in survival between aphids reared on rape and faba bean (Mann-Whitney *U* test, *p* < 0.001).

Aphid performance was evaluated with regard to its survival, development, and fecundity. Almost all (99%) *M. persicae* survived on rape by the end of the experiment, but the survival rate of *M. persicae* reared on faba bean decreased steadily until only 9% remained (Figure 1A). The survival rate of *M. persicae* reared on rape was significantly higher than that of *M. persicae* reared on faba bean (Mann-Whitney *U* test, *p* < 0.001). Moreover, the developmental time of *M. persicae* reared on rape was significantly shorter than that of *M. persicae* reared on faba bean (Mann-Whitney *U* test, *p* = 0.002; Table 2). The newly developed *M. persicae* adults reared on rape were significantly heavier than those reared on faba bean (Mann-Whitney *U* test, *p* < 0.001; Table 3). The fecundity of *M. persicae* reared on rape and faba bean was 27.8 ± 0.7 and 0, respectively (Table 4). Overall, the results suggest that rape and faba bean are suitable and poor hosts for *M. persicae*, respectively.

### 3.3. Performance of A. pisum

The performance of *A. pisum* was also evaluated through the measurement of its survival, development, and fecundity. Approximately 80% of *A. pisum* survived on faba bean by the end of the experiment (Figure 1B). By contrast, the survival rate of *A. pisum* reared on rape decreased sharply on the first day, and by day 2, all had dead (Figure 1B). The survival rate of *A. pisum* reared on faba bean was significantly higher than that of *A. pisum* reared on rape (Mann-Whitney *U* test, *p* < 0.001). The developmental time of *A. pisum* reared on faba bean was 6.0 ± 0.1 days, whereas all of *A. pisum* reared on rape died before developing into adults (Table 2). The newly developed *A. pisum* adults reared on faba bean was weighed 1.59 ± 0.06 mg on average (Table 3). Regarding fecundity, the faba bean-reared *A. pisum* produced 18.5 ± 1.0 progenies, but the rape-reared *A. pisum* did not survive long enough to reproduce (Table 4). Overall, the results suggest that faba bean and rape are a suitable host and a nonhost for *A. pisum*, respectively.

### 3.4. Probing and Feeding Behavior of M. persicae

**Figure 2:**
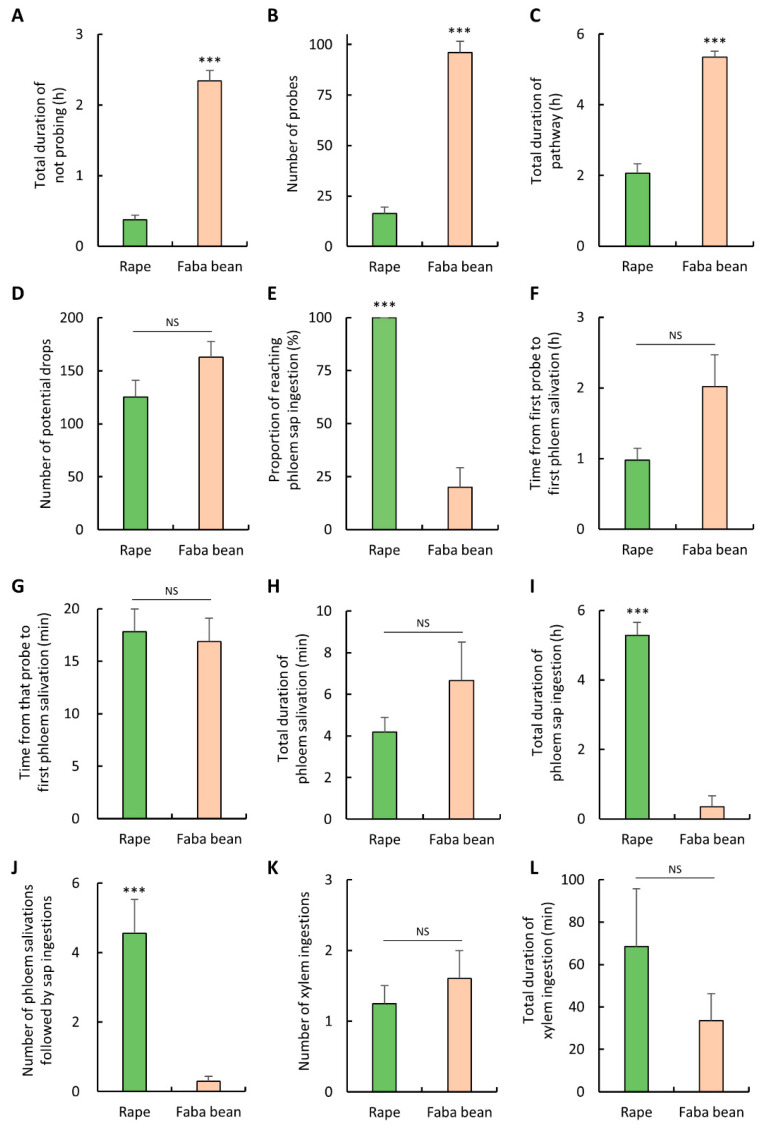
Waveform events and waveform duration of the (**A**–**D**) non-phloem parameters, (**E**–**J**) phloem-related parameters, and (**K**,**L**) xylem-related parameters of *M. persicae* on rape and faba bean. Error bars represent the standard errors of the means. Asterisks indicate significant differences between aphids probing on rape and faba bean (*** *p* < 0.001). NS indicates non-significant difference (*p* > 0.05).

To compare the probing and feeding behavior of *M. persicae* on rape and faba bean, probing and feeding waveforms were monitored using the EPG and then analyzed. Significant differences were detected among non-phloem parameters, with the exception of the number of potential drops (Figure 2A–D). Compared with the individuals on rape, *M. persicae* on faba bean spent significantly more time not probing (Mann-Whitney *U* test, *p* < 0.001; Figure 2A) and performed significantly more probes (Mann-Whitney *U* test, *p* < 0.001; Figure 2B). When probing on faba bean, the duration of pathway phase of *M. persicae* was significantly longer (*t* test, *p* < 0.001; Figure 2C); however, no significant difference in the number of potential drops was detected between *M. persicae* on rape and on faba bean (*t* test, *p* = 0.091; Figure 2D).

Even though 100% of *M. persicae* on rape achieved phloem sap ingestion, only 20% of those on faba bean achieved phloem sap ingestion within the 8-h EPG monitoring period (Figure 2E). *M. persicae* on faba bean had a significantly smaller proportion of successful phloem sap ingestion (χ^2^ test, *p* < 0.001; Figure 2E). The process of locating phloem tissues was assessed using the duration of the first phloem salivation from the first probe and from the beginning of that probe. No significant difference in the time from first probe to first phloem salivation was detected between *M. persicae* on rape and on faba bean (Mann-Whitney *U* test, *p* = 0.039; Figure 2F). Moreover, no significant difference was detected in the time from that probe to first phloem salivation between *M. persicae* on rape and on faba bean (Mann-Whitney *U* test, *p* = 0.557; Figure 2G). When the aphid stylet penetrates into the phloem tissue, the aphid first salivates to phloem and then ingests the phloem sap. Even though no significant difference was detected in the duration of phloem salivation between *M. persicae* on rape and on faba bean (Mann-Whitney *U* test, *p* = 0.517; Figure 2H), the number of phloem salivations followed by sap ingestions of *M. persicae* on faba bean was significantly lower than that of *M. persicae* on rape (Mann-Whitney *U* test, *p* < 0.001; Figure 2J). Furthermore, total duration of phloem sap ingestion of *M. persicae* on faba bean was significantly shorter than that of *M. persicae* on rape (Mann-Whitney *U* test, *p* < 0.001, Figure 2I). No significant difference was detected in the number and total duration of xylem ingestions between *M. persicae* on rape and on faba bean (Mann-Whitney *U* test, *p* = 0.730; Figure 2K; *t* test, *p* = 0.252; Figure 2L).

**Figure 3:**
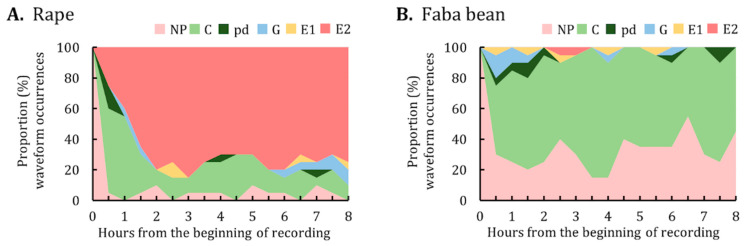
Proportion of waveform occurrences for *M. persicae* on (**A**) rape and (**B**) faba bean. The graph represents the percentage of individuals in each waveform performed by *M. persicae* at the end of each 30-min interval during the 8-h EPG monitoring period. NP, not probing; C, pathway; pd, potential drop; G, xylem ingestion; E1, phloem salivation; E2, phloem sap ingestion.

The proportion of waveform occurrences at the end of each 30-min interval over the 8-h EPG monitoring period was calculated and plotted (Figure 3). *M. persicae* on rape began to ingest phloem sap as early as 0.5 h after having access to plants. Overall, approximately 70–80% of the aphids performed phloem-related activities (i.e., E1 and E2) from hours 2–8 of the EPG monitoring (Figure 3A). By contrast, the major activities of *M. persicae* on faba bean were not probing and pathway activities; phloem-related activities were minimal (Figure 3B).

### 3.5. Probing and Feeding Behavior of A. pisum

**Figure 4:**
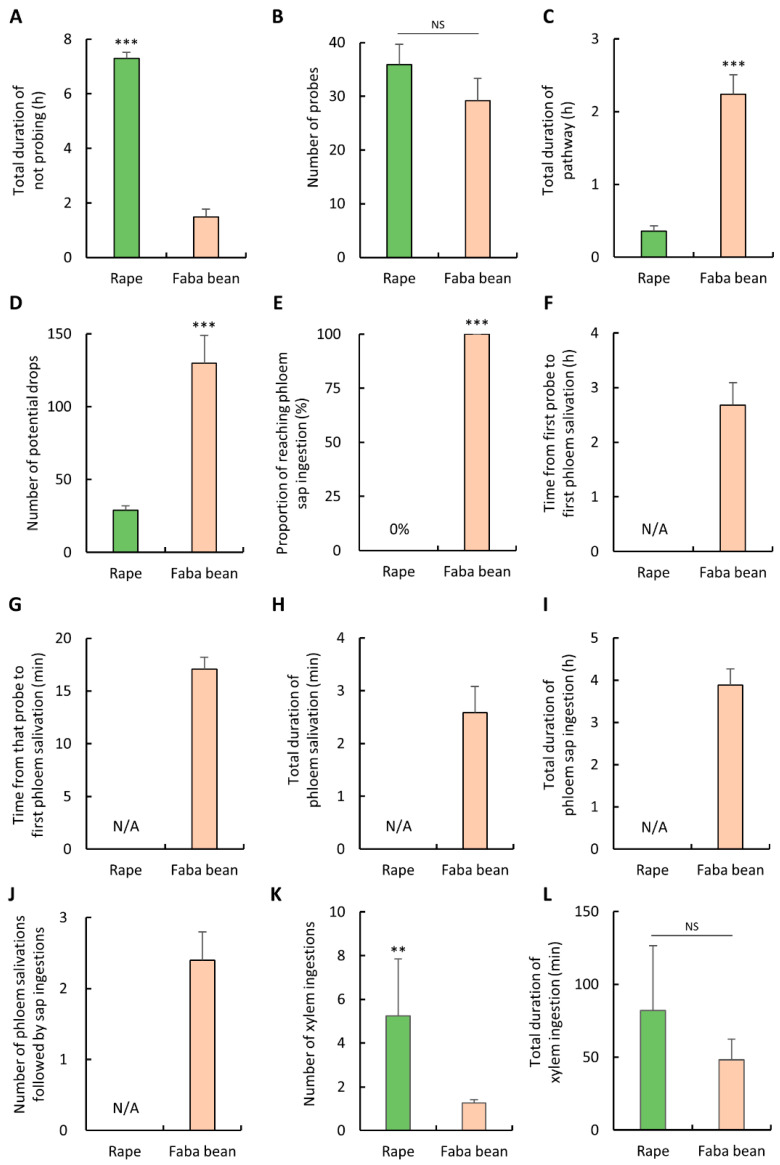
Waveform events and waveform duration of the (**A**–**D**) non-phloem parameters, (**E**–**J**) phloem-related parameters, and (**K**,**L**) xylem-related parameters of *A. pisum* on rape and faba bean. Error bars represent the standard errors of the means. Asterisks indicate significant differences between aphids probing on rape and faba bean (** *p* < 0.01; *** *p* < 0.001). NS indicates non-significant difference (*p* > 0.05).

To compare the probing and feeding behavior of *A. pisum* on rape and faba bean, probing and feeding waveforms were monitored using the EPG and then analyzed. Significant differences were detected among the non-phloem parameters, with the exception of the number of probes (Figure 4A–D). Compared with the individuals on faba bean, *A. pisum* on rape spent significantly more time not probing (Mann-Whitney *U* test, *p* < 0.001; Figure 4A), accounting for almost the entire EPG monitoring period. No significant difference in the number of probes was detected between *A. pisum* on rape and on faba bean (*t* test, *p* = 0.232; Figure 4B). *A. pisum* on rape had significantly shorter pathway phase (Mann-Whitney *U* test, *p* < 0.001; Figure 4C) and performed significantly fewer potential drops than *A. pisum* on faba bean (Mann-Whitney *U* test, *p* < 0.001; Figure 4D).

Notably, 100% of *A. pisum* on faba bean achieved phloem sap ingestion; however, no *A. pisum* on rape reached the phloem in the 8-h EPG monitoring period (Figure 4E). *A. pisum* on faba bean spent approximately 2.7 h from first probe to first phloem salivation (Figure 4F) and approximately 17 min from that probe to first phloem salivation (Figure 4G). On average, *A. pisum* on faba bean engaged in phloem salivation for less than 3 min (Figure 4H) and then engaged in prolonged phloem sap ingestion for 3.9 h (Figure 4I). The average number of phloem salivations followed by sap ingestions of *A. pisum* on faba bean was 2.4 (Figure 4J). When probing on rape, a nonhost plant, *A. pisum* engaged in significantly more xylem ingestion events (Mann-Whitney *U* test, *p* < 0.01; Figure 4K), although no significant difference in the total duration of xylem ingestion was detected (*t* test, *p* = 0.372; Figure 4L).

**Figure 5:**
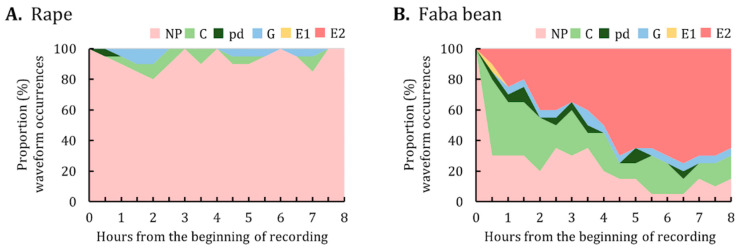
Proportion of waveform occurrences for *A. pisum* on (**A**) rape and (**B**) faba bean. The graph represents the percentage of individuals in each waveform performed by *A. pisum* at the end of each 30-min interval during the 8-h EPG monitoring period. NP, not probing; C, pathway; pd, potential drop; G, xylem ingestion; E1, phloem salivation; E2, phloem sap ingestion.

Data on the proportion of waveform occurrences indicated that the major activities of *A. pisum* on rape were not probing (Figure 5A). By contrast, *A. pisum* on faba bean began to ingest phloem sap (i.e., E2) as early as 0.5 h after having access to plants, with the proportion of phloem sap ingestion increasing over the 8-h EPG monitoring period (Figure 5B).

## 4. Discussion

*M. persicae* preferred rape to faba bean, whereas *A. pisum* preferred faba bean to rape. We further examined aphid performance on these plants and discovered that *M. persicae* survived and reproduced on rape; on faba bean, by contrast, reproduction was unsuccessful and few individuals (9%) survived. Therefore, rape is a suitable host and faba bean is a poor host for *M. persicae. A. pisum* survived and reproduced on faba bean but not on rape. Therefore, faba bean is a suitable host and rape is a nonhost for *A. pisum.*

The performance of *M. persicae* reared on rape and faba bean displayed differences in survival rate, developmental time, adult body weight, and fecundity. Even though *M. persicae* is a generalist species, it developed faster and yielded larger adult progenies on rape. By contrast, on faba bean, most *M. persicae* (91%) died within 5 days and could not reproduce. In one study, *M. persicae* reared on oilseed rape (suitable host) had a larger body size than *M. persicae* reared on barley (poor host), and its population size was eight times larger after 14-days rearing [13]. Francis et al. [10] compared the net reproductive rate (*R*_0_) of *M. persicae* reared on oilseed rape, white mustard (*Sinapis alba*), and faba bean and discovered that the lowest and highest *R*_0_ were observed on faba bean and oilseed rape, respectively. Another generalist species, *Aphis craccivora,* developed slowly and exhibited low fecundity and intrinsic rate of increase when reared on its non-preferred hosts [31].

Approximately 80% of *A. pisum* reared on faba bean survived, whereas all *A. pisum* reared on rape died within 2 days. We observed *A. pisum* nymphs wandering in the gauze bag on day 1, but they did not feed on the rape. By day 2 all *A. pisum* reared on rape had died. *A. pisum* reared on faba bean developed and reproduced well. In one study, *A. pisum* performs best in terms of body weight and survival rate on faba bean than on other legume species [8]. *A. pisum* reared on faba bean also exhibited longer reproductive period, higher fecundity, and higher survival rate compared with *A. pisum* reared on red clover (*Trifolium pratense*) and dwarf bean [9]. Aphids cannot survive and reproduce on their nonhosts. Examples include *Rhopalosiphum padi,* which was unable to survive and reproduce on *Arabidopsis* [14], and *Myzus cerasi,* which was unable to survive on barley [13]. These examples demonstrate the antibiosis resistance of nonhost plants to aphid species.

Distinct probing and feeding behaviors of *M. persicae* on rape versus on faba bean were revealed through EPG analysis. Regardless of whether an aphid encounters host or nonhost plants, it always conducts test probing [16,17,22,24]. *M. persicae* on faba bean performed more probing attempts than *M. persicae* on rape; the pathway phase was also longer. This probing is associated with the transmission of plant viruses during both host and nonhost interactions [4,17,25,26]. On a suitable host, the aphid stylet can puncture the phloem to facilitate sap ingestion [32,33]. The probing and feeding events are associated with the acquisition and inoculation of phloem-restricted viruses [4,17,27,28]. The number of phloem salivations followed by sap ingestions when *M. persicae* probed on rape was higher than when it probed on faba bean; moreover, the duration of phloem sap ingestion was longer. By contrast, few *M.* *persicae* (20%) on faba bean ingested the phloem sap, and their phloem sap ingestion duration was shorter than was that on rape. In addition, no significant differences were noted in the “time from first probe to first phloem salivation” and in the “time from that probe to first phloem salivation” between *M. persicae* on rape and on faba bean. This suggests that although the stylet of *M. persicae* easily moves through the mesophyll tissue, *M. persicae* has difficulty ingesting the phloem sap of faba bean. In one study, the EPG was applied in examining the probing and feeding behavior of *M. persicae* on *Arabidopsis* (suitable host) and barley (poor host) [22]. Similar to *M. persicae* on faba bean, *M. persicae* on barley performed more probes, fewer salivation events, shorter phloem salivation, and shorter phloem sap ingestion than on *Arabidopsis* [22].

The EPG analysis revealed that compared with *A. pisum* on faba bean, *A. pisum* on rape performed fewer potential drops, spent more time not probing, and exhibited a shorter pathway phase. As we observed in the aphid performance experiment, no *A. pisum* fed on rape. This was evident by none of the individuals was able to reach the phloem of rape. Therefore, no phloem-related parameters for *A. pisum* on rape could be calculated. The chemical cues controlling host acceptance of aphids are detected during the stylet penetration process [16]; thus, nonhost resistance is likely residing in the mesophyll tissues. *A. pisum* on rape performed more xylem ingestion events than did *A. pisum* on faba bean. Studies have reported that the occurrence and duration of xylem feeding in aphids increases following starvation period [34,35,36]. In this study, the stylet of *A. pisum* could not reach the phloem of rape. Thus, the aphids starved and became dehydrated, which in turn resulted in the increased occurrence of xylem ingestion. Aphids often ingest xylem sap [22,24,34,36,37], which potentially contributes to their water supply and osmoregulation [34,38]. The EPG has also been applied to study the probing and feeding behavior of *A. pisum* clone P1 on pea and faba bean (two suitable hosts) and alfalfa (a nonhost) [8]. *A. pisum* clone P1 on pea and faba bean exhibited more repetitive sieve element (SE) puncture and SE feeding periods, while no SE punctures and SE feeding were observed for the individuals on alfalfa [8].

*M. persicae* performed better and fed more successfully on rape than on faba bean. As a generalist species, *M. persicae* feeds on a broad range of host plants, and host plant choice depends on nutritional cues [16]. Rape and other brassicas harbor glucosinolate compounds [39,40], plant secondary metabolites that act as deterrents to various arthropod species [40,41,42]. Despite the high glucosinolate content in rape, *M. persicae* accepted rape as a suitable host. Similar results were reported in a study wherein the probing and feeding behavior of *M. persicae* were unaffected by various levels of glucosinolates produced by oilseed rape cultivars [43]. In contrast to *M. persicae*, the specialist *A. pisum* did not feed on rape, as indicated by its 100% mortality and the EPG experiments. This result is consistent with those of other investigations in which, *A. pisum* did not feed on faba bean artificially infused with sinigrin, a glucosinolate [44,45]. *Brevicoryne brassicae*, another specialist aphid, is unaffected by the glucosinolate-myrosinase defense system of brassicas [46]. These results demonstrated that the sequestration of glucosinolates from host plants is species-specific and may have as-yet-unknown evolutionary implications.

Conversely, *A. pisum* performed better and fed more successfully on faba bean than on rape. Unexpectedly, the generalist *M. persicae* neither performed nor fed well on faba bean. Medina-Ortega and Walker [11] demonstrated that phloem proteins called forisomes in faba bean can occlude SE and consequently inhibit the ingestion of phloem sap by *M. persicae*. However, the feeding of *A. pisum* on faba bean did not trigger such SE occlusion [47]. This contributes to various hypotheses. One posits that faba bean recognizes a putative elicitor in the saliva of *M. persicae*, and the elicitor triggers forisome occlusion. Another hypothesis states that a specific effector in the saliva of *A. pisum* has capacity to suppress the calcium channels responsible for triggering forisome occlusion [11].

Capable of reproducing parthenogenetically, aphids produce many offspring in a short time, and their ability to transmit a wide array of plant viruses compels the study of aphid-plant interactions from a broader perspective. Specialist and generalist aphids have differing impacts on the epidemiology of plant viral diseases. On host plants, the prolonged feeding of aphids follows test probing, with phloem salivation and sap ingestion causing the transmission of semipersistent, circulative-nonpropagative, and circulative-propagative viruses [4,17,27,28]. The feeding of *M. persicae* on various hosts has the capacity to transmit viruses that infect various plant species. When aphids land on poor hosts (e.g., when *M. persicae* lands on faba bean), they perform test probing and have a long pathway phase, but few achieve phloem sap ingestion. In such scenarios, the transmission of nonpersistent viruses by aphids is promoted. When aphids land on nonhosts (e.g., when *A. pisum* lands on rape), they perform a similar number of probes as they do when landing on host plants. However, compared with those on host plants, these aphids have a shorter pathway phase, perform fewer potential drops, and do not achieve phloem sap ingestion. In this case, most probing occurs on the epidermis that promotes the transmission of nonpersistent viruses [4,17,25,26]. This knowledge can be leveraged in developing integrated pest management packages against aphid-transmitted viral diseases.

## 5. Conclusions

*M. persicae* survived and reproduced well on rape, but few individuals survived on faba bean. *A. pisum* survived and reproduced well on faba bean, but none survived on rape. The EPG results demonstrated the feeding preferences of these two aphid species. *M. persicae* on faba bean performed more probes and exhibited a longer pathway phase, but few achieved phloem sap ingestion. *A. pisum* on rape performed a similar number of probes as *A. pisum* on faba bean did. However, *A. pisum* on rape had a shorter pathway phase and performed fewer potential drops, with none achieving phloem sap ingestion. The EPG results suggest that the resistance mechanisms of faba bean to *M. persicae* and those of rape to *A. pisum* are likely residing in the phloem and mesophyll tissues, respectively. Due to these distinct probing and feeding behaviors, specialist and generalist aphids have distinct impacts on the epidemiology of plant viral diseases.

## Figures and Tables

**Table 1:** Percentage of *Myzus persicae* and *Acyrthosiphon pisum* that settled on rape and faba bean.

Plant	*M. persicae*		*A. pisum*	
	1 h	3 h	1 h	3 h
Rape	87% a	90% a	12% b	5% b
Faba Bean	4% b	1% b	37% a	34% a

Different letters within the same column indicate a significant difference (Mann-Whitney *U* test, *p* < 0.05).

**Table 2:** Developmental time from first-instar nymph to adult of *M. persicae* and *A. pisum* reared on rape versus on faba bean.

Aphid	Developmental Time (Days, Mean ± SE)				
	*n*	Rape	*n*	Faba Bean	*p*
*M. persicae*	98	5.1 ± 0.0	2	7.5 ± 1.5	0.002
*A. pisum*	0	N/A	74	6.0 ± 0.1	N/A

Data were compared using the Mann-Whitney *U* test; N/A, not applicable.

**Table 3:** Body weight of newly developed *M. persicae* and *A. pisum* adults reared on rape versus on faba bean.

Aphid	Body Weight (mg, Mean ± SE)				
	*n*	Rape	*n*	Faba Bean	*p*
*M. persicae*	98	0.48 ± 0.01	2	0.20 ± 0.03	<0.001
*A. pisum*	0	N/A	74	1.59 ± 0.06	N/A

Data were compared using the Mann-Whitney *U* test; N/A, not applicable.

**Table 4:** Fecundity of *M. persicae* and *A. pisum* reared on rape versus on faba bean.

Aphid	Progenies (Mean ± SE)			
	*n*	Rape	*n*	Faba Bean
*M. persicae*	93	27.8 ± 0.7	2	0
*A. pisum*	0	N/A	71	18.5 ± 1.0

N/A, not applicable.

## Data Availability

The data presented in this study are available in Supplementary Table.

## Supplementary Materials

The following are available online at www.mdpi.com/2075-4450/12/11/975/s1.
